# Ki67 and the apparent diffusion coefficient in postoperative prostate cancer with endocrine therapy

**DOI:** 10.3389/fsurg.2023.1140883

**Published:** 2023-04-05

**Authors:** Jun He, Bangwei Che, Po Li, Wei Li, Tao Huang, Peng Chen, Miao Liu, Guangyu Li, Siwen Zhong, Kaifa Tang

**Affiliations:** ^1^ Department of Urology, The Affiliated Hospital of Guizhou Medical University, Guiyang, China; ^2^ Department of Pathology, The Affiliated Hospital of Guizhou Medical University, Guiyang, China; ^3^ Department of Urology and Andrology, The First Affiliated Hospital, Guizhou University of Traditional Chinese Medicine, Guiyang, China

**Keywords:** Ki67, ADC, prostate cancer, prognosis, endocrine therapy

## Abstract

**Background:**

Prostate-specific antigen (PSA) is a critical part of prostate cancer (PCa) screening, diagnosis, staging, and prognosis. However, elevated PSA levels can also be caused by several external factors. To improve the specificity and sensitivity of PSA in clinical practice, we explored whether markers or parameters may be used as prognostic targets for PCa by long-term follow-up.

**Methods:**

A total of 121 PCa patients who underwent laparoscopic radical prostatectomy (LRP) were included in our study, all of whom underwent imaging and preoperative pathological diagnosis. Endocrine therapy has long been applied to treat postoperative patients. The prognosis of enrolled patients was followed, and statistics were collected. Spearman's correlation analysis was applied to examine the relationship and clinical parameters. Kaplan–Meier analysis was used to process the clinical variables of PCa patients. Cox proportional hazards regression analysis was applied to examine univariate and multivariate variables.

**Results:**

The Gleason score (GS), PSA, clinical stage, nerve infiltration, organ confinement, Ki67 and apparent diffusion coefficient (ADC) were significantly associated with prognosis (all *P *< 0.05). The GS, PSA, clinical stage, organ confined, Ki67, nerve infiltration and ADC were included in the multivariate analysis (all *P *< 0.05). Ultimately, Ki67 and the ADC were found to provide meaningful predictive information (both *P *< 0.05).

**Conclusions:**

Ki67 and the ADC may be clinically and analytically valid prognostic biomarkers and imaging parameters in PCa. They may be useful for predicting the prognosis and risk of PCa patients undergoing postoperative routine endocrine therapy.

## Introduction

Prostate cancer is one of the most common urological malignancies in elderly men. Its morbidity and mortality rank second and fifth, respectively, among all malignant tumors worldwide (1). Currently, PSA (prostate-specific antigen), PSA derivatives, proPSA, and the prostate health index are being used in diagnostic practice (2). In particular, PSA is the most widely used oncological marker for screening, diagnosis, staging and prognosis (3). However, PSA has some limitations in clinical practice: a number of external factors can influence elevated PSA values, and high PSA levels cannot distinguish between aggressive and nonaggressive disease (4, 5). These limitations may lead to overdiagnosis and subsequent overtreatment combined with limited access to PSA testing and therapeutic response in PCa (1). Therefore, it is important to identify molecular markers that represent the biological activity of malignant prostate cells. In recent years, Ki67 has attracted increased study interest as a prognostic, predictive, and potentially therapeutic target for malignant cancers (6, 7).

Ki67, a nuclear DNA-binding protein expressed in all vertebrates, is connected to cell proliferation activity (8). Ki67 is present throughout the whole cell cycle except the resting and G0 phases (6). The Ki67 labeling index and positive incidence can be detected using IHC on paraffin-embedded sections, which can assist urologists in formulating effective therapy regimens and optimizing follow-up schedules for patients with prostate cancer (9). Imaging examination—particularly magnetic resonance imaging (MRI), which also offers a method to quantitatively analyze water diffusion in PCa tissue—is an important functional technique for diagnosis (10–12). In particular, the ADC may act as a useful differential definition by delivering reliable information for the GS of suspected prostate cancer (10). The ADC has also been shown to be helpful for classifying and identifying lesions as well as evaluating disease activity (13, 14).

Over the past decade, the efficacy and safety of LRP have improved, thus providing a minimally invasive alternative to open surgery with shorter recovery times and hospital stays (15, 16). More recently, robot-assisted laparoscopic prostatectomy (RALP) has become another noninvasive panoramic approach (17). Of note, the core of systemic therapy is testosterone reduction. Endocrine therapy for PCa remains a cornerstone after radical prostatectomy (18). In particular, androgen deprivation therapy (ADT) is well considered to have more potential benefits (19).

Currently, most studies have been conducted on the predictive and clinical effectiveness of Ki67, but few studies have utilized the characteristics of Ki67 and the ADC in combination. We strongly believe in the importance of ADCs in PCa and hold the opinion that Ki67 can be applied in combination with postoperative PSA testing and imaging parameters (i.e., the ADC) to improve specificity and sensitivity (9, 20, 21). The aim of this study was to determine whether Ki67 had any predictive value for prostate cancer patients who received surgery and endocrine therapy.

## Subjects and methods

### Human subjects

This study protocol and informed consent form were approved by The Affiliated Hospital of Guizhou Medical University (Guiyang, China), and written and verbal informed consent was obtained from all patients. Data from the retrospective study were collected from 2013 to 2022, and 149 patients who underwent radical prostatectomy were included in our research. All patients with PCa were included in this study based on the following criteria: (1) Written and verbal informed consent was obtained from all patients before participation in the study. (2) Prostate cancer was pathologically confirmed by prostate biopsy. (3) Those patients' physical conditions were relatively good, and the patients' life expectancy was greater than 10 years. (4) Prostate cancer is usually localized (up to T3b). The exclusion criteria for all PCa patients were as follows: (1) severe cardiopulmonary insufficiency or crucial organ dysfunction; (2) uncorrected coagulation disorders; (3) severe mental disorder or cognitive impairment; (4) poor nutritional status; and (5) history of previous radiation or immunomodulation therapy. All patients were followed up until June 30, 2022, and the end point event was the death of patients due to PCa. During a median 56 months of follow-up, a total of 28 patients were excluded from our study, including 7 patients who were lost to follow-up due to telephone changes or address changes, 11 patients who died of other causes, 6 patients who developed castration-resistant prostate cancer, and 4 patients who received additional chemotherapy and immunotherapy.

### MRI protocols

**Figure 1:**
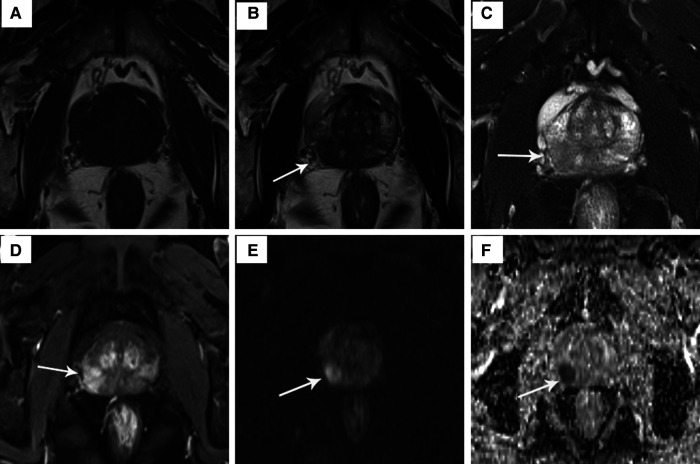
The signal characteristics of a 64-year-old PCa patient on T1W image, T2W image, T2W enhancement image, DWI and ADC map. (**A**) T1W image: no obvious discovery; (**B**) T2W image: mild hypointensity in the prostate's intermediate right posterolateral zone (arrow); (**C**) hypointensity on T2W fat suppression sequence; (**D**) T2W enhancement image: hyperintensity confirmed presence of lesion; (**E**) hyperintensity on DWI (*b* = 800 s/mm^2^); (**F**) hypointensity on ADC map.

All patients were examined using a 3.0-T human MRI scanner (Siemens, Erlangen, Germany) in the supine head-first position combined with a phased-array surface coil. T1-weighted (T1W) imaging was performed in the axial plane with the following parameters: repetition time (TR), 500.00 ms; echo time (TE), 9.00 ms; matrix 225 × 200; and slice thickness, 3.50 mm. Axial T2-weighted (T2W) imaging was performed with the following parameters: TR, 3,500 ms; TE, 108 ms; matrix, 225 × 200; and section thickness, 3.50 mm. Axial T2W imaging enhancement was observed with the following parameters: TR, 3.94 ms; TE, 1.48 ms; matrix, 200 × 230; and section thickness, 2.00 mm. Diffusion-weighted imaging (DWI) was acquired using the single-shot echo-planar imaging technique with three different b values (0, 800, and 1,600 s/mm^2^). The apparent diffusion coefficient (ADC) value was calculated by monoexponential regression, and ADC mapping was automatically generated pixel by pixel using all b values. The mean ADC was 1.038 × 10^−3^ mm^2^/s in this study (Figure 1).

### Sample collection and analysis

Serum PSA levels were measured one week before LRP, and PCa samples were collected using laparoscopic radical resection. Complete prostatectomy and bilateral seminal vesicles were performed during surgery. During the surgery, the lymph nodes, including the obturator lymph nodes, internal iliac lymph nodes, external iliac lymph nodes, and common iliac lymph nodes, were dissected. PCa samples and lymphoid tissue were used to make the final pathological diagnosis.

### IHC for Ki67

The surgically removed tissue samples were dehydrated in accordance with the dehydration protocol using an automated dehydrator after already being fixed with 4% paraformaldehyde. Prostate cancer samples were completely embedded in paraffin and sliced into 5-µm-thick sections. Slices were baked at 60 degrees for 1 h, followed by dewaxing and hydration. After antigen retrieval and removal of endogenous peroxidase, nonspecific epitopes were blocked by immunostaining blocking solution at room temperature. Sections were stained overnight with rabbit anti-human primary antibody (Abcam, Cambridge, UK), and sections were stained with goat anti-rabbit secondary antibody (Abcam, Cambridge, UK). Hematoxylin and 3,3-diaminobenzidine (DAB) were utilized to stain tissue sections separately. After immersing the sections in various concentrations of graded ethanol, they were covered with coverslips. The sections were observed and photographed using a microscope.

### IHC analysis

**Figure 2:**
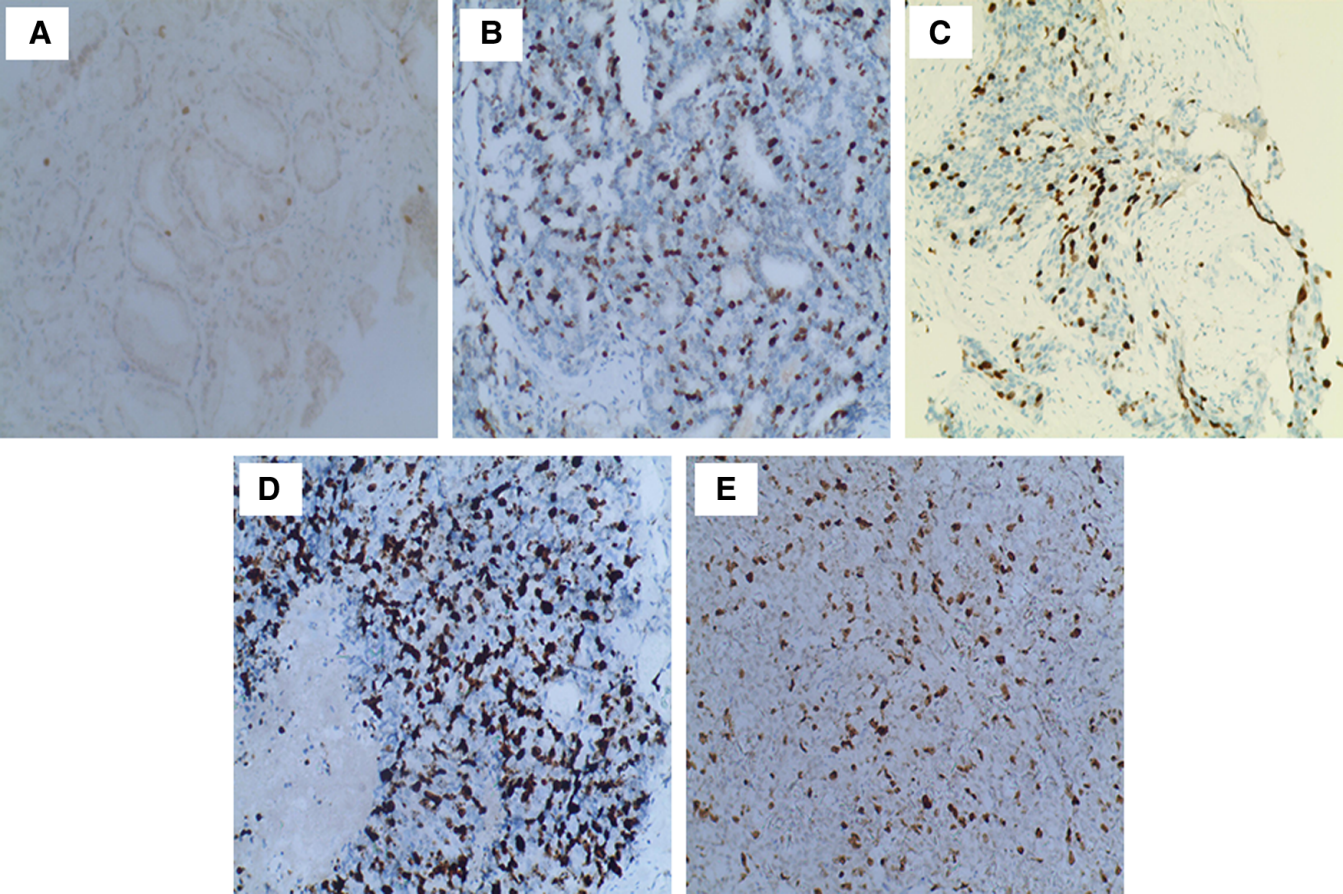
Immunohistochemical staining of postoperative prostate cancer specimens for Ki67. (**A**) Ki67 expression for GS 6: only a few tumor cells stained brownish-yellow nuclei (100×); (**B**) Ki67 expression for GS 7 (4 + 3, 100×); (**C**) Ki67 expression for GS 8 (4 + 4, 100×): rising brown–yellow staining in nuclei; (**D**) Ki67 expression for GS 9 (5 + 4, 100×); (**E**) Ki67 expression for GS 10 (100×): strongly brown–yellow expression in nuclei.

The expression of Ki67 in prostate cancer samples was assessed independently by two pathologists. IHC slides were photographed, and the positive cells were formally counted as the Ki67 labeling index. The results were divided into four groups based on the percentage of Ki67-positive cells (7). Cases in which the percentage of Ki67-stained cells was ≤5% were considered 1+. Cases with a Ki67 labeling index of 5%–25% were considered 2+, and 26%–50% were considered 3+. Cases with more than 50% Ki67-stained cells were considered 4+ (Figure 2).

### Postoperative endocrine therapy

The endocrine therapy of lutein hormone-releasing hormone (LHRH) agonist coupled with nonsteroidal antiandrogen drugs was acceptable by all patients. Goserelin sustained-release implants (AstraZeneca UK Limited, UK), one of the LHRH agonists, were injected subcutaneously at 28-day intervals. Bicalutamide (Corden Pharma GmbH, Germany) is recommended as one tablet daily (50 mg/day). We conducted long-term follow-up with patients once every month to ensure therapy compliance, evaluate effectiveness, and detect adverse events. The mean duration of endocrine therapy was 41 months, with a maximum duration of 72 months.

### Statistical analysis

Statistical analysis was performed with Statistical Product and Service Solutions (SPSS 24.0, IBM, Chicago, IL, United States). The quantitative data were expressed as the mean ± standard deviation (SD), and the quantitative data were statistically classified as either qualitative or ordinal data. Spearman correlation was applied to test the relationship between Ki67 expression and clinical parameters. Kaplan‒Meier analysis was used to process univariate variables of prostate cancer patients. Cox proportional hazards regression analysis (Forward:LR) was applied to examine multivariate variables. A Cox proportional hazards regression model was used for proportional hazards analysis. All *P* values <0.05 were considered statistically significant, and *P* values were two-tailed. Survival curves were drawn using GraphPad Prism 9.0 (GraphPad Software, San Diego, California, United States).

## Results

### Demographic and clinical characteristics of patients

Table 1 shows the demographic and clinical characteristics of the PCa patients included in the study. The mean age ± standard deviation at the time of surgery was 70.45 ± 7.29 years. Almost 96% of patients had a body mass index (BMI) over 18.5. Based on the analysis of histopathological specimens, GS ranged from 6 to 10. The mean PSA ± standard deviation was 5.72 ± 4.02 with a median PSA value of 3.80 ng/ml. The pathological tumor stage of these surgical specimens was categorized according to morphological characteristics from pathology reports. The numbers of patients with unilateral PCa, bilateral PCa and extraprostatic PCa were 40, 40 and 26, respectively. Ki67 expression was 1+ for 46 cases, 2+ for 43 cases, 3+ for 21 cases and 4+ for 11 cases. Sixty-two PCa cells with nerve infiltration were discovered. Thirty-eight patients had positive surgical margins. In addition, preservation of the neurovascular bundle (NVB) was unilateral in 42 cases and bilateral in 57 cases. The ADC was divided into two groups based on the average.

**Table 1:** Clinical characteristics of PCa patients.

Clinical characteristics	Cases
**Age (years)**	
<70	52
≥70	69
**BMI (kg/m^2^)**	
<18.5	5
18.5–24.0	77
>24.0	39
**GS**	
6	29
7	40
8	15
9	24
10	13
**PSA (ng/ml)**	
<4	64
4–10	40
>10	17
**Clinical stage**	
≤T2	80
T3	41
**Organ confined**	
Unilateral	40
Bilateral	40
Extraprostatic extension	26
Others	15
**Ki67 (%)**	
<5%	46
5%–25%	43
25%–50%	21
>50%	11
**Nerve infiltration**	
Yes	62
No	59
**Surgical margin**	
Positive	38
Negative	83
**Preservation of NVB**	
Unilateral	42
Bilateral	57
No preservation	22
**AUC**	
≤1.038 × 10^−3^ mm^2^/s	52
>1.038 × 10^−3^ mm^2^/s	69

BMI, body mass index; GS, Gleason score; PSA, prostate-specific antigen; NVB, neurovascular bundle; AUC, apparent diffusion coefficient.

### Correlations of Ki67 expression and the ADC with clinical characteristics of PCa patients

**Figure 3:**
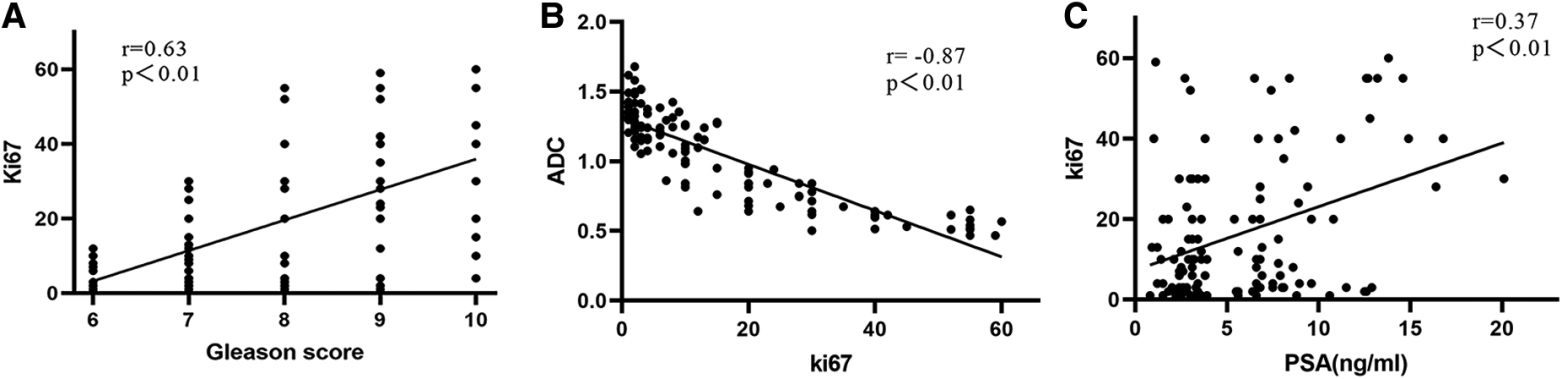
Correlations in different Ki67 expression levels in PCa patients. (**A**) GS and Ki67; (**B**) Ki67 and ADC; (**C**) Ki67 and PSA value.

The correlations of PCa patients are listed in Table 2. Ki67 expression was significantly correlated with GS (*r *= 0.514, *P *< 0.01), PSA (*r *= 0.248, *P *< 0.05), clinical stage (*r *= 0.281, *P *< 0.05), organ confinement (*r *= 0.349, *P *< 0.01), and nerve infiltration (*r *= 0.251, *P *< 0.05). Spearman correlation analysis revealed that the ADC was significantly correlated with GS (*r *= −0.461, *P *< 0.01), PSA (*r *= −0.195, *P *< 0.05), nerve infiltration (*r *= −0.416, *P *< 0.01), and surgical margin (*r *= −0.315, *P *< 0.01). Ki67 expression was positively correlated with GS (*r *= 0.630, *P *< 0.01) and PSA (*r *= 0.370, *P *< 0.01) (Figures 3A,C) and significantly negatively correlated with the ADC (*r *= −0.870, *P *< 0.01) (Figure 3B).

**Table 2:** Correlations of clinical characteristics of PCa patients.

Clinical Variables	Ki67		ADC	
	*r*	*P*	*r*	*P*
GS	0.514	<0.01	−0.461	<0.01
PSA	0.248	<0.05	−0.195	<0.05
Clinical stage	0.281	<0.05	−0.144	0.115
Organ confined	0.349	<0.01	−0.268	0.065
Nerve infiltration	0.251	<0.05	−0.416	<0.01
Surgical margin	0.154	0.215	−0.315	<0.01
Preservation of NVB	0.107	0.441	0.217	0.217

GS, Gleason score; PSA, prostate-specific antigen; NVB, neurovascular bundle.

### Survival analysis of PCa prognostic factors

**Figure 4:**
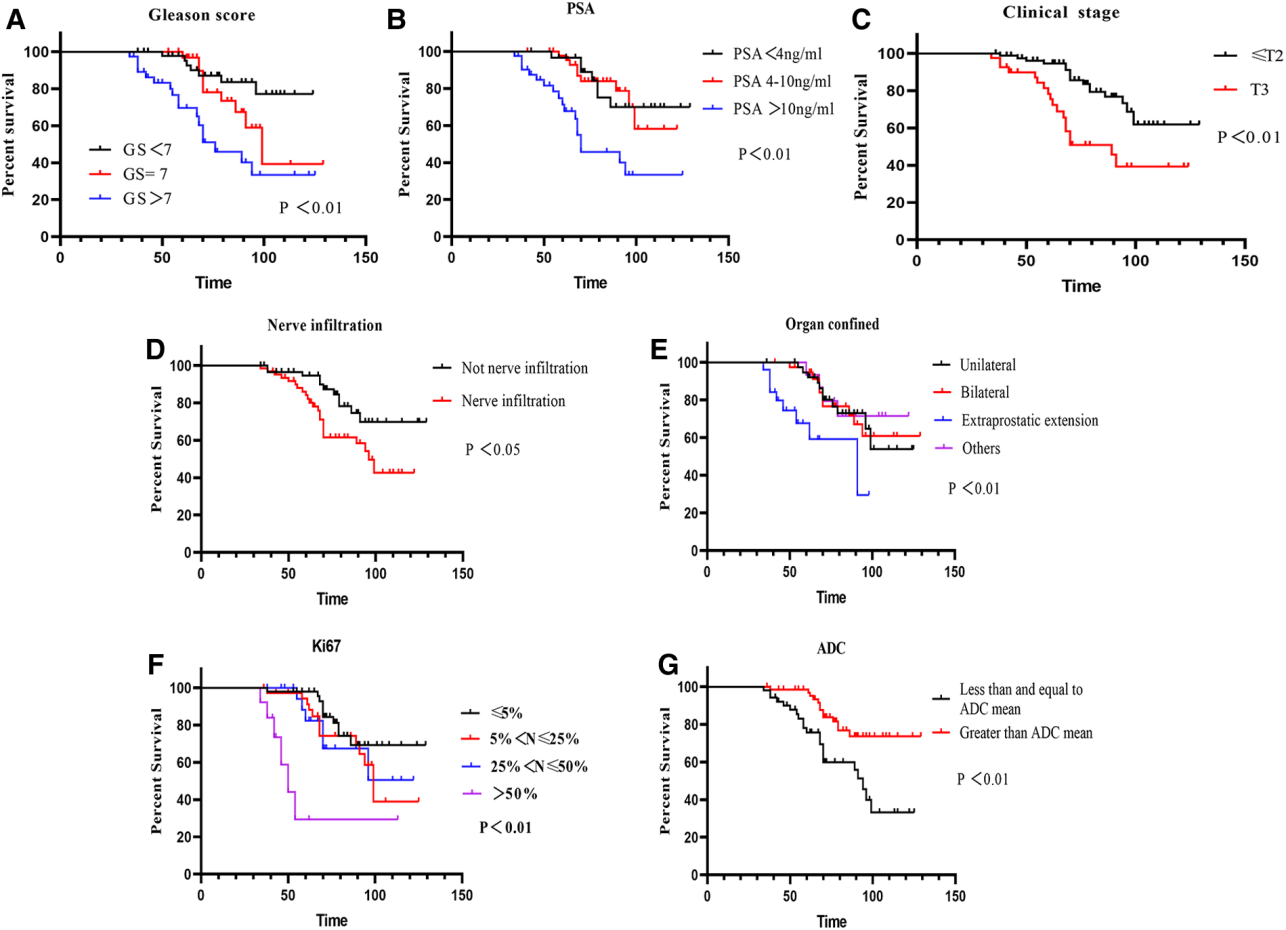
Survival curves of PCa patients influencing prognosis. (**A**) Survival curves of GS (*P *< 0.01); (**B**) survival curves of PSA (*P *< 0.01); (**C**) survival curves of clinical stage (*P *< 0.01); (**D**) survival curves of nerve infiltration (*P *< 0.05); (**E**) survival curves of organ confined (*P *< 0.05); (**F**) survival curves of Ki67 (*P *< 0.01); (**G**) survival curves of ADC (*P *< 0.01).

As shown in Figure 4, Kaplan‒Meier analysis was employed to identify clinical variables that may affect prostate cancer prognosis. In long-term follow-up patients after laparoscopic radical prostatectomy, GS, PSA, clinical stage, nerve infiltration, organ confinement, Ki67 and the ADC were significantly associated with prognosis (Figures 4A–G, all *P *< 0.05), which were significantly identified as more adverse variables. In univariate analysis, significant differences were discovered by Cox proportional hazards regression analysis. GS, PSA, clinical stage, organ confined, Ki67, nerve infiltration and the ADC were included in the multivariate analysis (Table 3, all *P *< 0.05). In multivariate analysis, Ki67 [HR = 1.61, 95% CI (1.06–2.43)] and the ADC [HR = 0.99, 95% CI (0.31–1.79)] may provide meaningful predictive information (Table 3, both *P *< 0.05).

**Table 3:** Cox regression analysis of PCa patients.

Clinical variables	Univariate analysis		Multivariate analysis	
	HR (95% CI)	*P*	HR (95% CI)	*P*
GS	1.59 (0.98–2.36)	<0.01	1.42 (0.85–2.06)	0.06
PSA	1.27 (0.61–2.18)	<0.01	1.21 (0.58–1.85)	0.09
Clinical stage	2.62 (1.54–3.94)	<0.05	2.61 (1.49–3.55)	0.12
Organ confined	1.70 (1.12–2.58)	<0.05	1.52 (0.99–2.37)	0.18
Ki67	1.78 (1.24–2.55)	<0.01	1.61 (1.06–2.43)	<0.01
Nerve infiltration	2.24 (1.09–4.62)	<0.05	2.15 (1.01–4.13)	0.21
Surgical margin	1.85 (0.91–3.56)	0.09		
Preservation of NVB	1.29 (0.71–2.24)	0.11		
ADC	1.06 (0.37–1.98)	<0.01	0.99 (0.31–1.79)	<0.05

HR, Hazard Ratio; CI, confidence interval.

### Cox proportional hazards analysis of PCa prognostic factors

Cox regression multiplicative interaction analysis was applied to analyze three variables. Ki67 and ADC revealed that clinical variables satisfied proportional hazards.

## Discussion

The World Health Organization (WHO) most recent research estimates that PCa in men is diagnosed in nearly 1.4 million new cases and 375,000 deaths worldwide (1). With the growth of the aging population, the increasing incidence of prostate cancer has already drawn attention, whereas mortality rates are gradually decreasing (1). The global burden of socioeconomic inequality and the cost of health care are both growing rapidly. Currently, PSA is the most widely used cancer biomarker for prostate cancer screening. However, PSA is susceptible to other factors in urinary disorders, such as age, acute prostatitis, ejaculation, and catheterization, and thus lacks sensitivity and specificity (2, 5). Enhancing clinical management is crucial for detecting prostate cancer early and predicting its aggressiveness and prognosis (3, 5).

Recent studies have discovered that Ki67 can be used as a diagnostic and prognostic biomarker in renal cancer (6), breast cancer (20, 22), ovarian cancer (23), craniopharyngioma (24) and neuroendocrine neoplasms (25). However, the prognostic role of Ki67 in prostate cancer remains ambiguous and has not been thoroughly explored. Therefore, our study intends to shed light on the significance of Ki67 as a potential biomarker in conjunction with imaging and pathology in diagnosis, as well as to investigate its prognostic potential in prostate cancer by long-term follow-up data. In this study, we evaluated the significant positive correlation of Ki67 with GS, and we observed significant survival differences with clinical stage, nerve infiltration and Ki67 expression. We also found a significant difference in Ki67 expression levels using Cox regression analysis. These findings suggest that Ki67 is a prognostic marker in postradical prostatectomy prostates.

There remains a lack of clarity regarding the clinical application and health care of PSA. Indeed, PSA screening has a variety of benefits for the elderly population and gives them the opportunity to detect disease in its early stages (19, 26). On the other hand, PSA screening also results in overdiagnosis; that is, 40% of men diagnosed with indolent prostate cancer may never show any clinical symptoms (27, 28). Additionally, PSA screening can also induce many adverse effects, such as anxiety from false-positive PSA tests and complications from further investigation (26). Our study revealed no significant differences in different PSA levels when using survival analysis, raising slight doubts about PSA screening. The review also suggested that PSA screening prefers to identify men who may harbor clinically significant disease (29).

Over the past two decades, multiparametric MRI (mp-MRI) containing T1W imaging, T2W imaging and DWI has gained widespread acceptance in the management of PCa patients because of its high accuracy (12, 30). Evidence from a high-quality study showed that MRI scanning prompts one in four men to avoid biopsy and reduces the detection of nonaggressive PCa (31). T2WI, which was adopted as a reference, had higher pooled sensitivity and specificity when DWI and the ADC were combined (30, 32). Recent studies have demonstrated that a high level of suspicion of DWI and the ADC on MRI was closely associated with the identification of PCa, indicating that the combined application of these sequences and quantitative metrics enables the visualization of suspicious lesions (33, 34). Our study also supports their conclusions. Consequently, the ADC is calculated and has been adopted for evaluating multiple and large lesions (13). MRI-based ADC and T2WI scans showed good performance in malignant prostate lesions and predicting extracapsular extension and positive surgical margins (35).

More recently, minimally invasive techniques have taken the place of surgical treatments for prostate cancer (17). In particular, RALP has become an additional surgical option for prostate cancer patients. All patients in this study underwent laparoscopic radical prostatectomy. In men with localized prostate cancer, PSA concentrations should fall to a relatively low level (<0.1 μg/L) within 2 months of successful radical prostatectomy (36). The Gleason grading system was employed to score all postoperative samples, and pathological sections of Ki67-stained immunosections were observed and photographed in accordance with the IHC protocol. The Gleason score is integral to the clinical management of prostate cancer and has been used for over 50 years as one of the closest prognostic indicators based on the histological appearance of prostate cancer cells (37, 38). Previous studies have shown a substantial connection between GS in PCa and long-term prognosis (39, 40). In this study, we also found a significant correlation between Ki67 and GS, suggesting that Ki67 can be used as a prognostic indicator.

Ki67, a nuclear DNA binding protein originating from its city of name (Kiel) and the 67th original clone in the 96-well plate, is a biomarker of proliferation to evaluate the proportion of dividing cells to grade tumors (41). Previous research has revealed that Ki67 expression levels are relatively higher in the G1, S and G2 phases from cell cycle analysis (6). The GS in prostate cancer was found to be strongly correlated with Ki67 expression in our study, and it was suggested that this relationship, along with other prostate cancer prognostic factors, can be used to assess prognostic significance (7). In addition, a recent study demonstrated that increasing antigen retrieval is a helpful tool for outdated pathology archives, potentially extending the preservation time of PCa tissue samples (42). In our results, a significant difference in Ki67 expression was found by survival analysis and Cox proportional hazards analysis, which is consistent with their study. Furthermore, Ki67 acts as a good molecular surrogate and therapy response for prognosis (6, 43). It is well known that ADT brings more potential benefits after radical prostatectomy (19). ADT, inhibited by eliminating androgens as an endocrine therapy, is the cornerstone of prostate cancer treatment. The study revealed that ADT was synergistic with radiation therapy (RT) in all patients with locally advanced PCa (18, 44). Based on the pulsatile release of LHRH from the hypothalamus, other approaches to regulating androgen production by manipulating LHRH were developed for clinical use (45). LHRH agonists were prescribed daily to prostate cancer patients who had their serum testosterone levels suppressed by 75% and their plasma acid-phosphatase levels decreased or normalized (45). The aforementioned regimens were administered to PCa patients in our study over an extended period of time. Treatment strategies involving the proliferation biomarker Ki67 have shown promising results (6, 22). In this study, endocrine therapy represented a significant advancement for Ki67 perspectives. As these endocrine tools advance, urologists will be better able to tailor treatments to individual PCa patients and improve their outcomes.

Although the follow-up duration was long in the current study, more patients should be included in future studies. Potential or relative variables may not have been identified due to small sample sizes. Additionally, a few variables are included because some data are either missing or inadequate. In addition, the single-center design in our study was a limitation, and multicenter studies are required for further clarification.

## Conclusions

Ki67 and the ADC are highly associated with PCa patients who undergo laparoscopic radical prostatectomy. The assessment of proliferation-associated nuclear protein Ki67 *via* IHC and the assessment of ADC *via* MRI may provide prognostic biomarker and imaging parameters with analytical and clinical validity in PCa. For PCa patients with postoperative routine endocrine therapy, Ki67 and the ADC are prognostic factors that bring new approaches.

## Data Availability

The original contributions presented in the study are included in the article/Supplementary Material, further inquiries can be directed to the corresponding author/s.
